# Integrating Neurology, Palliative Care and Emergency Services in ALS: A Community-Integrated Neuropalliative Pathway in Modena, Italy

**DOI:** 10.3390/brainsci15121294

**Published:** 2025-11-30

**Authors:** Gianfranco Martucci, Sofia Charis Bonilauri, Alberto Canalini, Marcello Baraldi, Luigi Costantini, Fabio Mora, Paolo Vacondio

**Affiliations:** 1 Rete Locale di Cure Palliative, AUSL Modena, 41121 Modena, Italy; 2 Specializzazione in Cure Palliative, Università di Modena e Reggio Emilia, 41121 Modena, Italy; 3 Department of Emergency, AUSL Modena, 41121 Modena, Italy

**Keywords:** amyotrophic lateral sclerosis, palliative care, integrated care, advance care planning, case study, community health

## Abstract

Background: Amyotrophic lateral sclerosis (ALS) is a progressive neurodegenerative disease that causes severe motor, respiratory and communication impairment and imposes a high psychosocial burden on patients and families. Recent evidence shows that integrated neuropalliative care—early collaboration between neurology and palliative services with community support—improves quality of life and reduces avoidable hospitalisations. Yet there are few descriptions of how such integration is operationalised. Objective: This study examines a Community-Integrated Neuropalliative Pathway (CINP) implemented in the province of Modena (Emilia-Romagna, Italy), analysing how neurology, palliative care and emergency services collaborate to provide continuous, person-centred care for people with ALS. Methods: A single, holistic case study was conducted following Yin’s analytical approach. Data sources included ten semi-structured interviews with neurologists, palliative physicians, nurses, home-care professionals and emergency clinicians; ethnographic observations in the ALS outpatient clinic; relevant organisational documents (the regional Clinical Pathway on ALS); and aggregated quantitative data from the palliative care registry (January 2023–December 2024). Thematic analysis with investigator triangulation was used to explore care integration, advance care planning and emergency coordination. Quantitative data were summarised descriptively. Results: Three interrelated themes were identified: (1) Progressive and flexible integration between neurology and palliative care. Neurologists remained longitudinal reference points while palliative teams were activated in response to evolving needs and became more relevant with the progression of the disease. Regular multidisciplinary meetings and shared discharge planning facilitated coordination. (2) The shared culture of advance care planning. Professionals framed advance care planning (ACP) as a relational, iterative process anchored in therapeutic relationships. Shared care plans, once completed, triggered an electronic Emergency Warning (“warning 118”) procedure that notified the emergency service of patient preferences. (3) The integration of palliative and emergency services. The warning system enabled emergency clinicians to respect care plans and avoid aggressive interventions during crises. Quantitative data on 47 ALS patients followed by territorial palliative services showed that 16 had an active Emergency Warning flag; among these, most died at home or in a hospice rather than in hospital. Conclusions: The Modena CINP exemplifies how a public health system can operationalise early neuropalliative integration and connect hospital, community and emergency services. The qualitative findings illustrate the cultural and organisational shifts required for continuous care, while the quantitative data show that the system is correctly used and that patients with the Emergency Warning activation died mostly at home or in a hospice. Lessons from this analytical case study can inform the development of similar pathways in other regions, although further research is needed to assess outcomes in larger populations and such models need to be adapted to local contexts.

## 1. Introduction

Amyotrophic lateral sclerosis (ALS) is a rare yet devastating neurodegenerative disease characterised by the progressive loss of motor neurons. Patients gradually develop severe muscle weakness and respiratory failure and usually die within three to five years. The burden of ALS extends beyond physical decline: patients experience dysphagia, dyspnoea, communication difficulties and often cognitive or behavioural changes. Families face emotional distress, caregiving demands and financial strain [1]. As the disease progresses, decisions about ventilation, feeding tubes and end-of-life care become increasingly complex; comprehensive care must therefore address physical symptoms, psychosocial support, ethical considerations and future planning [2].

The international literature emphasises the need for a multidisciplinary and person-centred approach to ALS management, integrating neurology with respiratory, nutritional, psychosocial and ethical expertise [1,3]. Systematic reviews and cohort studies show that early palliative involvement improves symptom control, facilitates shared decision-making and reduces emergency admissions [4,5,6]. Nevertheless, neuropalliative models remain unevenly implemented: in many settings palliative care is introduced late, sometimes only after respiratory failure, due to misconceptions that palliative care equates to “giving up” or because of fragmented health systems. Cross-sectional surveys report that clinicians recognise the value of palliative care but cite limited access and unclear referral pathways as barriers [7] and dying in their preferred place is often difficult for ALS patients.

Organisational case studies might help to illuminate how integrated neuropalliative pathways are implemented in practice and how they can bridge hospital-based neurology with community services and emergency systems [8,9].

In the Emilia-Romagna region (Italy), the regional health authority developed a Clinical Pathway (Percorso Diagnostico Terapeutico Assistenziale, PDTA) for ALS that mandates integrated management across hospital and territorial services [10]. The Local Health agency of Modena developed a local version of the PDTA in 2016, which specifies that care should be shared between general practitioners, neurologists, respiratory and nutrition specialists and territorial teams, with the goal of continuity across pre-hospital, hospital and post-hospital phases and socio-sanitary integration [11]. The ALS PDTA laid the foundation for a hub and spoke model with the neuromuscular clinic at the University Hospital of Modena as the hub and community services as spokes.

Building on this policy, the province of Modena developed a **Community-Integrated Neuropalliative Pathway (CINP)** through the years. This pathway aims to engage neurology, palliative care, home-care and the Emergency Warning (“Warning 118”) in a coordinated network that supports advance care planning and respects patients’ preferences during crises.

A distinctive feature of the CINP is in fact the Emergency Warning procedure: once a patient completes a shared advance care plan (SCP, in Italian Pianificazione Condivisa delle Cure, PCC) and consents, a coordination nurse uploads key information into a shared file accessible to the 911 (Italian 118) command centre. When a call comes from or about that patient, emergency operators can review the plan and align their interventions with the stated wishes. This innovation reflects the pathway’s broader ambition to connect hospital neurology, palliative care and territorial services, fostering continuity between elective and emergency care. However, there are no clear organisational models in the scientific literature.

To help address this gap, we conducted an analytical case study of the Modena CINP, focusing on how professionals experience and implement integration across settings and exploring preliminary quantitative indicators of pathway impact.

In 2021–2022 a participatory action research project coordinated by the local health authority (AUSL Modena) developed a protocol to link palliative care teams and emergency services (the Emergency Warning procedure), resulting in an organisational report and a published article [12]. That research focused on developing the procedural framework and evaluating initial perceptions.

Building upon that work, the present article undertakes an analytic case study [13] to examine how the Community Integrated Neuropalliative Pathway has been formalised and how it functions in practice. The analytic case study aims to generate theoretical insights that extend beyond the local context—rather than seeking statistical generalisation, it uses the Modena case to refine understandings of the organisational mechanisms needed for cross-setting integration. Reporting follows the consensus standards for organisational case studies [14], which emphasise the explicit description of the case, the rationale for case selection, data sources, analysis methods and interpretive rigour.

## 2. Methods

### 2.1. Study Design

We adopted a single, holistic case study design with an analytical purpose, as described by Yin [13]. In this approach, cases are chosen not to achieve statistical generalisation but to illuminate theoretical propositions and generate transferable insights. Our unit of analysis was the organisational pathway itself (CINP), constituted of different protocols and documents, comprising neurology, palliative care, home-care and emergency services. We sought to examine how this network functions to deliver continuous, person-centred care for ALS patients. Analytical case studies enable an in-depth understanding of complex interventions in real-world contexts and are particularly suited to examining healthcare organisations [14].

### 2.2. Case Selection and Context

The CINP is operated by the Azienda Unità Sanitaria Locale (AUSL) Modena in partnership with the Azienda Ospedaliero-Universitaria (AOU) of Modena. The AUSL delivers community and territorial health services and “spoke” hospital care, while the AOU provides “hub” specialist hospital care, including the ALS outpatient clinic.

The pathway covers a population of approximately 700,000 residents in the Modena province. The regional PDTA for ALS, issued by Emilia-Romagna in 2013, mandates coordination between general practitioners, neurologists and territorial services and emphasises integrated care across pre-hospital, hospital and post-hospital phases [10]. We considered as part of the CINP the whole pathway of the patient, which includes outpatient management in the practice of the University Hospital in the early stages, the referral of all ALS patients to home palliative care when more complex needs emerge, multidisciplinary meetings every three to four months, shared advance care planning with patients and families, and the activation of the Emergency Warning procedure.

### 2.3. Use of the Term “Community”

In this article, the term community is used in the organisational sense, consistent with the international public health and primary care literature. It refers to the network of territorial health services as opposed to hospital-based services or civic voluntary associations. This clarification is important because “community-based palliative care” can also denote compassionate community movements or volunteer-led initiatives.

### 2.4. Participants and Data Collection

#### 2.4.1. Qualitative Component

We used purposeful sampling to include diverse professional perspectives from the pathway. Between July and September 2025 we conducted ten semi-structured interviews with two neurologists (including the ALS clinic director), one palliative physician, two palliative care nurses, a home-care nurse, a physician and three nurses from the emergency services. Interviews lasted 0–60 min and were audio-recorded and transcribed verbatim. Topics included experiences of collaboration, the timing of palliative involvement, processes of advance care planning, the use of the Emergency Warning system and perceived barriers and facilitators (see the interview guide in Supplementary Material, File S1).

We also recorded non-participant observations during ALS outpatient sessions to document interactions among professionals and between clinicians and patients/families. Observations focused on communication dynamics, decision-making and the integration of services. In addition, we reviewed organisational documents including the PDTA, standard operating procedures for the Emergency Warning and meeting minutes.

#### 2.4.2. Quantitative Component

To complement the qualitative insights, we extracted aggregated registry data for all ALS patients followed by AUSL Modena’s territorial palliative care services from January 2023 to December 2024. For each patient we recorded whether the Emergency Warning procedure had been activated, reasons for activation, vital status at 31 December 2024 and place of death. Data were anonymised and analysed descriptively.

### 2.5. Data Analysis

#### Qualitative Analysis

Two authors independently read the transcripts and field notes and conducted inductive thematic analysis using open coding. Codes were organised into preliminary themes and then refined through constant comparison and discussion into three overarching categories, reflecting the integration between neurology and palliative care, advance care planning culture and palliative–emergency collaboration. Triangulation across interviewers, observations and documents enhanced credibility. Disagreements were resolved by consensus. Reflexive memos documented analytic decisions and researcher assumptions. The analysis followed recommendations for rigour in organisational case studies [14]. The researchers that conducted the qualitative analysis kept a focus on personal reflexivity by trying to be aware of their own biases during the process (see Supplementary Material, File S2).

### 2.6. Quantitative Analysis

We summarised the counts and percentages for the activation of the warning procedure and for outcomes (alive, deceased; place of death; main reason for the call) in the registry data. Given the small sample and observational design, statistical testing was not appropriate; the results are considered descriptive and exploratory.

## 3. Results

### 3.1. Qualitative Themes

#### 3.1.1. Progressive and Flexible Integration Between Neurology and Palliative Care

Professionals consistently described the integration between neurologists and palliative teams as the cornerstone of the CINP. Neurologists remained the primary point of reference throughout the disease trajectory, but palliative involvement was tailored to individual needs rather than tied to a specific stage. One neurologist explained the following:

“We remain the main reference along the entire course of the disease; patients appreciate that we decide when to activate the palliative team. Sometimes activation happens very late—only at the final stage—when something hasn’t worked, but this is rare now.” (int. 1)

A palliative care professional agreed, “Over time, coordination has improved, and we are working together in a more timely manner” (int. 10).

This flexible approach contrasts with models where palliative care is introduced only at the end stage. Professionals credited regular multidisciplinary meetings (every three months) with enabling the early discussion of complex cases. A palliative nurse noted the following:

“Now we have regular joint meetings where patients seen in neurology are reported in advance. We prepare discharge plans together, so everyone knows which patients will go home and what kind of home assistance will be required.” (int. 2)

Such meetings facilitated shared decision-making and reduced fragmentation. Participants emphasised that integration occurred not only in formal meetings but also through informal contacts and the possibility to reach colleagues at any time. Ethnographic observations showed that in some outpatient sessions, multidisciplinary physical presence remained limited, with communication primarily between the patient and the neurologist, in the role of the main case manager.

#### 3.1.2. Building a Shared Culture of Advance Care Planning

A central innovation of the CINP is the emphasis on advance care planning (ACP). Professionals described ACP not as a single document but as a relational process rooted in trust and repeated conversations. A palliative nurse explained the following:

“We hold meetings at the patient’s home together with neurologists and the palliative team; we explore the issues and jointly decide on the path to take. The shared care plan is written only after several encounters, when the patient fully understands.” (int. 3)

The facilitator of the plan varies depending on who has the strongest therapeutic bond: this is sometimes the palliative doctor, sometimes the neurologist, other times a nurse. Professionals criticised the assumption that only palliative specialists should lead advance planning. They emphasised that ACP evolves through continuous awareness building rather than binary choices.

“Advance care planning is not about saying yes or no to certain treatments; it’s the endpoint of a long internal process—understanding the illness, its evolution and above all one’s own values.” (int. 4)

Once finalised, ACP triggers the Emergency Warning procedure: the coordination nurse uploads key information (patient identity, care plan, emergency instructions) into a shared file accessible to the 911 dispatch centre. This ensures that emergency health professionals are aware of patient preferences and can adapt their interventions accordingly. Families are described as feeling reassured by this mechanism, as it reduced the risk of unwanted hospitalisation or invasive procedures. At the same time, professionals underscored that ACP remains an evolving document; it can be revisited as the disease progresses or family circumstances change.

#### 3.1.3. Integration of Palliative and Emergency Services: The “Emergency Warning” System

The Emergency Warning procedure is an operational innovation linking palliative care with emergency services. When a call comes from a registered patient, the dispatcher sees a flag and accesses the care plan. An emergency health professional described the following:

“When we receive a call for a patient on the warning list, we already know the situation and can respect their wishes instead of applying standard protocols.” (int. 5)

This integration requires clear communication and trust. Emergency clinicians reported that, historically, they felt obligated to perform aggressive interventions (e.g., intubation) because they lacked information about patient preferences. The warning system allowed them to tailor responses and avoid morally distressing situations. Despite its benefits, participants identified challenges: a lack of fully interoperable digital records across hospitals and community services; the reliance on dedicated nurses to update the warning file; and limited 24-h palliative coverage for specialist palliative care consultation. Emergency staff advocated for continued training on neuropalliative principles.

Table 1 summarises a set of partially conflicting values that emerged in the neuropalliative interprofessional work for ALS care, highlighting the key tensions observed in our experience.

### 3.2. Quantitative Findings

During 2023–2024 the AUSL Modena territorial palliative services followed 47 patients with ALS. By 31 December 2024, 37 had died and 10 were alive. The Emergency Warning procedure had been activated for 16 patients (34%). Among those with an active warning: 15 had died (10 at home, 2 in a hospice, none in residential care and 3 in hospital), while 1 remained alive. Among the 31 patients without warning, 9 were alive; of the 22 deceased, 11 died at home, 1 in a hospice, 2 in residential care and 8 in hospital.

The main reason for hospital admission was breathlessness (see Box 1).

Box 1 **Quantitative findings.**

ALS patients 2024–2025 in home care: 47

Dead on 31 December 2025: 37

Patients with activated Emergency Warning 23–24: 16

Death place in patients without Emergency Warning:

10 at home

2 in hospice

3 in hospital

Death place in patients without Emergency Warning:

11 at home

1 in hospice

8 in hospital

2 in residential care

Reasons listed for hospital admissions:

29 hospitalizations:

Breathlessness (17)

Choice (3)

Anxiety (3)

Fever, epilepsy, fall, constipation, fatigue (1)

During this first period of implementation, the patients with warning activation died at home or in a hospice, avoiding a hospital death, more often than those without warning. However, given the small sample and observational design, no causal conclusions can be drawn. The findings should be interpreted cautiously but suggest that the Emergency Warning mechanism may support dying in the preferred setting.

An example of the expected user experience during the CINP is presented through clinical vignettes in Table 2.

## 4. Discussion

### 4.1. Integration of Hospital and Community Services

This case study sheds light on how an Italian health system operationalises the integration between neurology, palliative care and emergency services for ALS. The regional Clinical Pathway mandates collaboration across sectors but its enactment depends on local leadership and innovation. In Modena, the early involvement of the territorial palliative team and regular multidisciplinary meetings fostered continuity from diagnosis to end-of-life. Neurologists maintained longitudinal relationships while acknowledging the added value of palliative expertise. This contrasts with models where palliative care is introduced only at terminal stages or remains external to the neurology clinic. Our findings echo studies from Germany [15] and the United States [16] showing that embedding palliative specialists within ALS clinics or establishing home-based neuropalliative programmes enhances symptom management and caregiver satisfaction. Other Italian regions, such as Friuli Venezia Giulia, have developed clinical pathways that address the integration of the emergency services, but mainly on the topic of emergency life-preserving treatments [17].

The Modena CINP further extends integration by connecting hospitals and emergency services via the Emergency Warning procedure. Such trans-organisational coordination is rarely described in the current literature to the best of our knowledge, which highlights the potential of public health systems to align acute responses with personalised care plans.

### 4.2. Advance Care Planning as Relational Work

The pathway’s emphasis on relational advance care planning resonates with international calls to normalise early conversations about prognosis and preferences [18]. Participants distinguished ACP from bureaucratic forms, describing it as a process of building shared understanding and trust. This approach addresses ethical dilemmas such as the timing of tracheostomy or non-invasive ventilation and helps families prepare emotionally and logistically. The flexible role of the facilitator—whether the neurologist, nurse or palliative physician—underscores that advance planning is an interdisciplinary responsibility. By linking ACP to the Emergency Warning system, the CINP ensures that preferences documented in the community are respected during emergencies. Nevertheless, only one third of patients in the registry had an active warning flag. Future quality improvement efforts should monitor uptake and explore barriers to activation.

### 4.3. Transforming Emergency Care Culture

The interviews described the warning system as transformative: it shifted emergency services practice from applying standard life-saving protocols to aligning interventions with patient goals. This reduced moral distress and improved communication with families. These findings align with the literature on ethics in pre-hospital care, which emphasises the importance of advance directives and training in palliative communication for emergency providers [19,20]. However, the system relies on informational infrastructure and workforce capacity: updating the warning file requires dedicated staff; the Emergency Warning dispatchers need access to the database; and palliative teams must be available for consultation. Gaps in electronic interoperability and uneven coverage may limit scalability. Integrating the warning flag into regional electronic health records could enhance reliability.

### 4.4. Methodological Reflection

Analytical case studies are used for examining complex healthcare interventions. By focusing on a single, information-rich case, we were able to explore the interactions among multiple services, capture professional experiences and contextualise organisational innovations. Following reporting standards for organisational case studies [14], we clearly defined the case, described data sources and analysed the interplay of factors shaping the outcomes. The use of triangulation (interviews, observations, documents and registry data) strengthened validity.

Nevertheless, limitations must be acknowledged. The qualitative component involved a small number of participants; perspectives of patients, caregivers and general practitioners were not included, which may have provided additional insights. The Emergency Warning registry data covered only two years and lacked details on functional status or comorbidities, precluding a robust evaluation of outcome differences. We also lack data on why only one third of patients activated the Emergency Warning procedure.

Moreover, as an observational case study, the findings are not statistically generalisable. Rather, they offer analytic generalisation: transferable lessons about how integrated neuropalliative pathways can function within a public health system.

### 4.5. Comparison with Other Models and Implications

The Modena CINP shares features with home-based neuropalliative programmes in Germany [15] and the United States [16,21], such as early referral, multidisciplinary teams and an emphasis on advance care planning. However, its innovation lies in linking palliative and emergency services through a shared alert, reflecting a community-integrated perspective. For policymakers, the case study underscores that integrated care requires both structural arrangements (shared electronic systems, joint meetings) and cultural change (mutual respect, shared responsibility). For clinicians, the findings highlight the importance of relational continuity and early conversations. For researchers, this study points to the need for mixed-methods evaluations of neuropalliative pathways across different contexts and conditions.

We developed a checklist in the form of yes/no questions that summarises the key focus areas for organising a similar clinical pathway to support future stakeholders in addressing comparable organisational needs (see Table 3).

## 5. Conclusions

The Community-Integrated Neuropalliative Pathway in Modena demonstrates that integrated care for ALS is achievable within a public health system when neurology, palliative care, community services and emergency providers collaborate. Through the flexible activation of palliative support, relational advance care planning and an innovative Emergency Warning procedure, the pathway aligns care with patient preferences and may enable more people to die at home or in a hospice rather than in hospital. While this study’s qualitative insights and preliminary registry data are promising, further research should assess patient and caregiver outcomes, investigate factors influencing the uptake of the warning system and explore scalability to other regions and diseases. Nonetheless, this analytical case study provides a rich example of how organisational and cultural integration can transform care for people with serious illnesses.

## Figures and Tables

**Table 1:** Key value tensions in integrated neuropalliative care for ALS in the CINP case study.

Value 1		Value 2
Timing of decision-making		
Work on advance care planning while the patient can still communicate effectively	vs.	Introducing choices too early may cause stress-induced paralysis and global refusal
Respect for autonomy and awareness		
Avoid steering the patient’s decision	vs.	Concern that the patient does not grasp the severe implications of living with PEG and ventilation
Prognostic uncertainty		
Avoid “alarming” the patient given the uncertainty about disease evolution	vs.	Unpredictability can lead to rapid survival or communication issues
Life-sustaining interventions		
The burden of initiating life-sustaining support (e.g., PEG, NIV)	vs.	The burden of supporting the withdrawal of these supports when they become intolerable
Role of GP		
Value of GP support in daily management of potentially long-surviving patients	vs.	Difficulty for GPs to engage in highly specialised and ethically complex dynamics
Symptom relief and respiratory risk		
Use of morphine and benzodiazepines for symptom relief	vs.	Fear of suppressing respiratory function
Non-invasive ventilation (NIV)		
Use of NIV to palliate symptoms before definitive choices	vs.	Starting machine dependence that the patient might not desire after deeper reflection
Place of death		
Honour the patient’s wish to die in the preferred place	vs.	Complexity of symptom management for family caregivers at home
Long-term disability		
Potentially long phase of severe disability even without life-sustaining devices	vs.	Lack of support structures for disabilities of this magnitude (hospice stays limited; nursing homes reluctant)
Emergency interventions vs. reflective deliberation		
Need for clarity on intervention and legal/ethical protection during emergencies	vs.	Inherently slower pace of palliative reflections and deliberation

**Table 2:** Vignettes illustrating the integrated pathway (names and situations are fictional).

Stage	Patient Vignette	Provider/Family Perspective
Diagnosis and referral to integrated pathway	Giulia, 58, is diagnosed with ALS at the neuromuscular clinic. After a few months, her ability to walk is severely impaired, and she asks to have a conversation on how things are going to be in the future and what are her options. The neurologist explains the prognosis but also introduces the Community-Integrated Neuropalliative Pathway. Giulia learns that palliative care is not just for the end of life. She is offered an early consultation and told about the option to activate a shared care plan and the warning system when she feels ready.	The neurologist remains Giulia’s primary point of contact and schedules a multidisciplinary meeting to discuss her needs. Family members are relieved to know there is a coordinated pathway connecting home care, neurology and emergency services, and that decisions about interventions (e.g., PEG, NIV) can be discussed proactively.
Home palliative activation and needs assessment	Months later, Giulia experiences increasing fatigue and finds swallowing difficult. Her neurologist contacts the palliative team. A home nurse visits and performs a needs assessment. Giulia is now formally referred to the local palliative care network (LPCN). Regular home visits and social worker support begin.	The palliative team assesses Giulia’s needs and explains that the home team will coordinate with the rest of the team, including the emergency services if needed. Family is regularly involved in goals-of-care discussion.
Shared care planning and warning activation	During a series of meetings at home, Giulia, a palliative doctor and the home nurse discuss her values and preferences. They talk about her wishes regarding intubation, resuscitation and hospital admission. Often, her neurologist is present too. After several conversations, Giulia signs a shared care plan that specifies she does not want invasive ventilation and prefers to remain at home if possible. The nurse uploads the plan into the Emergency Warning system.	Professionals emphasise that advance care planning is relational and can be revisited over time; the document is not a one-off decision. The family receives training on how to call emergency services and inform them that a warning flag exists. They feel empowered by the process.
Emergency event with warning	Giulia develops respiratory distress at night. Her partner calls 118 and mentions that there is a warning flag. The dispatcher sees the flag, reads Giulia’s shared care plan and informs the ambulance crew. Paramedics arrive with action cards indicating “do not resuscitate” and “no hospital admission”. They provide symptom relief at home.	Emergency clinicians appreciate having clear instructions and avoid defaulting to invasive ventilation. Giulia’s family is relieved that the emergency response aligns with her wishes and that she can remain at home.
End-of-life care and bereavement support	Giulia’s condition declines further. She and her family reaffirm her wish to remain at home. The palliative team provides intensive home visits, symptom control and psychosocial support. She dies peacefully at home, surrounded by her family. After her death, the team offers bereavement support to her partner.	The home care team coordinates with the general practitioner to manage symptoms and medications. Family members express gratitude that Giulia’s preferences were honoured and that they received support during and after her death.
Emergency event without prior palliative flag	Luca, another ALS patient, has never been referred to the palliative team. He visits the ER for severe dyspnea. ED staff use the VAPU screening tool and identify palliative care needs. He is stabilised, and upon discharge the ED nurse contacts the LPCN within 48 h so the palliative team can evaluate him at home.	The ED nurse informs Luca’s caregiver about palliative options and arranges follow-up. This referral pathway reduces the risk of repeat emergency visits and ensures that Luca receives appropriate support.

**Table 3:** Checklist for integrated neuropalliative care pathways.

No.	Domain	Checklist Question
1	Leadership and Roles	Who initiates palliative care involvement (e.g., neurologist, palliative physician, general practitioner)?
2		Is there a defined case manager across the continuum of care?
3		Are there dedicated coordinators or nurses responsible for connecting home palliative care with emergency services?
4		Are emergency department (ED) professionals identified as palliative care champions or trained to provide primary palliative care?
5	Integration and Coordination	Are there regular multidisciplinary meetings (e.g., every three months) involving neurology, palliative care, home care and emergency services?
6		Is there a formal mechanism (e.g., “warning 118” or alert) to share advance care plans and patient preferences across hospital, community and emergency services?
7		Does the emergency department use a screening tool (e.g., VAPU) to identify patients with palliative care needs when not previously known to palliative services?
8		Are there clear procedures for ED staff to notify home palliative care teams upon discharge or admission?
9		Are referral pathways between ED and the local palliative care network well defined and documented?
10		Does the pathway align with regional/national clinical pathway (PDTA) guidelines?
11	Advance Care Planning	Is advance care planning viewed as a relational, iterative process rather than a one-time form?
12		Do patients and families have multiple meetings to develop and revisit their shared care plan?
13		Is the final care plan available to emergency systems for consultation (e.g., the warning 118) to ensure concordance during crises?
14		Does the care plan explicitly capture patient wishes regarding resuscitation, intubation, hospital admission and palliative sedation?
15		Are patients and families trained on how to contact the emergency service and communicate their status and preferences?
16	Education and Culture	Do ED professionals receive structured training in palliative care, including symptom management, communication skills and ethical issues?
17		Are there joint educational initiatives to build a shared culture of palliative care among the different professionals involved in assistance, as neurology, palliative, home care and emergency teams?
18		Are mechanisms in place to address bureaucratic delays, logistical challenges and potential resistance from practitioners?
19		Does your service’s training include non-cancer diagnoses like ALS, acknowledging their unpredictable trajectories?
20		Is there a continuous quality improvement process to gather feedback from staff and adapt the pathway and training accordingly?
21		Did you adopt an interprofessional and shared vision when developing the clinical pathway? Approaches such as action research or participatory research may be appropriate.
22	Quality Monitoring and Outcomes	Are indicators monitored (e.g., number of patients referred from ED to palliative care, number of flagged patients, place of death) to evaluate integration outcomes?
23		Is patient and caregiver satisfaction with the integrated pathway regularly evaluated?
24		Are end-of-life outcomes (e.g., home versus hospital death) recorded and used for quality improvement?
25		Are data and outcomes shared with stakeholders to promote accountability and improvements?
26		Is there flexibility to adapt the pathway to local contexts, recognising that no “one-size-fits-all” model exists?

## Data Availability

The data presented in this study are available from the corresponding author upon request, due to anonymity protection.

## Supplementary Materials

The following supporting information can be downloaded at: https://www.mdpi.com/article/10.3390/brainsci15121294/s1, File S1: topic_guide; File S2: reflexivity statement; File S3: Integrated Neuropalliative Care Attachments.
