# Supplementary material for: Integrating Neurology, Palliative Care and Emergency Services in ALS: A Community-Integrated Neuropalliative Pathway in Modena, Italy
Source: Brain Sci. 2025 Nov 30;15(12):1294. doi: 10.3390/brainsci15121294 (PMC12730195; doi:10.3390/brainsci15121294)
Supplement: Supplementary file 1 [file brainsci-15-01294-s001.zip › brainsci-4012585Attached Message Part/Attached Message Part]

\_\_\_\_\_\_\_\_\_\_\_\_\_\_\_\_\_\_\_\_\_

**Canalini Alberto**

Mobile +393393949165

E-mail: alberto.canalini@gmail.com

> Il giorno 28 nov 2025, alle ore 15:00, Brain Sciences Editorial Office <brainsci@mdpi.com> ha scritto:

> ﻿Dear Dr. Canalini,  
>   
> We invite you to proofread your manuscript to ensure that this is the final   
> version for publication and to confirm that you require no further changes:  
>   
> At MDPI, we believe in the fast dissemination of sound, valid scientific   
> knowledge. Once accepted for publication, we aim to ensure that research is   
> published as soon as possible.   
>   
> Please upload the final proofread version of your manuscript within 24 hours,   
> and please remember that we can be flexible with this timeframe if necessary.   
> If you need more time, please inform the Assistant Editor of the date by   
> which you will be able to return the proofread version.   
>   
> Please also ensure that you check that your manuscript has been properly   
> categorized by our AI tool during proofreading here:  
> https://category-connect.mdpi.com/brainsci-4012585/f0820fe5e57e74a7  
>   
> Our new AI tool (Category Connect), once launched, will assist with MDPI's   
> mission to foster open scientific exchange by enhancing the discoverability   
> of related papers on the MDPI website. We hope that you will assist us in the   
> development of this important tool by validating the categories identified   
> for your paper.   
>   
> Manuscript ID: brainsci-4012585  
> Type of manuscript: Article  
> Title: Integrating Neurology, Palliative Care and Emergency Services in ALS:   
> a Community‑Integrated Neuropalliative Pathway in Modena, Italy  
> Authors: Gianfranco Martucci, Sofia Charis Bonilauri, Alberto Canalini \*,   
> Marcello Baraldi, Luigi Costantini, Fabio Mora, Paolo Vacondio  
> Received: 12 Nov 2025  
> E-mails: gfmartucci@gmail.com, sofiacharis16@gmail.com,   
> alberto.canalini@gmail.com, ma.baraldi@ausl.mo.it,   
> luigi.costantini.mo@gmail.com, f.mora@ausl.mo.it, p.vacondio@ausl.mo.it  
> Neurorehabilitation  
> https://www.mdpi.com/journal/brainsci/sections/Neurorehabilitation  
> Palliative Care for Patients with Severe Neurological Impairment  
> https://www.mdpi.com/journal/brainsci/special\_issues/273I64FMO7  
>   
> Please read the following instructions carefully before proofreading:  
>   
> 1) Download the manuscript from the link provided at the end of this message   
> and upload the final proofed version via the second link. If you experience   
> any difficulties, please contact the Brain Sciences Editorial Office.   
>   
> 2) Please use Microsoft Word's built-in track changes function to highlight   
> any changes you make, or send a comprehensive list of changes in a separate   
> document. Note that this is the \*last chance\* to make textual changes to the   
> manuscript. Some style and formatting changes may have been made by the   
> production team. Please do not revert these changes.  
>   
> 3) All authors must agree to the final version. Carefully check that the   
> authors' names and affiliations are correct, and that funding sources are   
> correctly acknowledged. Incorrect author names or affiliations are picked up   
> by indexing databases, such as the Web of Science or PubMed, and can be   
> difficult to correct.  
>   
> After proofreading, final production will be carried out. Note that changes   
> to the position of figures and tables may occur during the final steps.   
> Changes can be made to a paper published online only at the discretion of the   
> Editorial Office.   
>   
>   
> Please download the final version of your paper for proofreading here:  
>   
> https://susy.mdpi.com/user/manuscripts/proof/file/392450100e02fa9d532dd62852eb707d  
>   
> and upload here:  
>   
> https://susy.mdpi.com/user/manuscripts/resubmit/392450100e02fa9d532dd62852eb707d  
>   
> We look forward to hearing from you soon.  
>   
> Kind regards,  
> Ms. Simone Liu  
> Special Issue Editor   
>   
> MDPI (Tianjin)  
> Floor 7, Block A, Lujiazui Financial Plaza,  
> Hongqiao District, 300131 Tianjin, China  
> Tel.: +86 22 2727 5507  
> -----------  
> MDPI   
> Brain Sciences Editorial Office  
> E-Mail: brainsci@mdpi.com  
> http://www.mdpi.com/journal/brainsci  
>   
> https://www.mdpi.com  
> Data Protection Notes: https://www.mdpi.com/about/data-protection  
> MDPI's headquarters are located in Basel, Switzerland. More information is   
> available here: https://www.mdpi.com/about/contact  
> Disclaimer: The information and files contained in this message are   
> confidential and intended solely for the use of the individual or entity to   
> whom they are addressed. If you have received this message in error, please   
> notify me and delete this message from your system. You may not copy this   
> message in its entirety or in part, or disclose its contents to anyone.
