# Supplementary material for: Integrating Neurology, Palliative Care and Emergency Services in ALS: A Community-Integrated Neuropalliative Pathway in Modena, Italy
Source: Brain Sci. 2025 Nov 30;15(12):1294. doi: 10.3390/brainsci15121294 (PMC12730195; doi:10.3390/brainsci15121294)
Supplement: Supplementary file 1 [file brainsci-15-01294-s001.zip › File 1 topic_guide.docx]

# Topic Guide – Interviews with Professionals (ALS, Palliative Care, Network, 118)

**Duration:** 30–45 minutes
**Format:** Semi‑structured
**Scope:** Internal evaluation (QI)
**Audio recording:** Optional (if authorised); otherwise, the interviewer will take notes.

## Opening Ethical Script (≈60 seconds)

“This interview is part of an internal evaluation/Quality Improvement project on the ALS pathway in the Province of Modena. The data will be analysed and presented anonymously and in aggregate form. Your participation is voluntary; you may decline to answer any question and may stop the interview at any time. May I record the audio? (Yes/No). Do you confirm your consent to participate?”

## Common Backbone (for all professional profiles)

### A. Warm‑up (≈2 minutes)

1. **Current role and years of experience.**
2. **Phases of the pathway:** At which stages do you most often meet ALS patients?

### B. Perception of the “Modena Model” (≈3 minutes)

1. **Practical distinction:** What, in practice, distinguishes the Modena pathway for ALS patients? (mention one strength and one challenge).

### C. Advance Care Planning (≈5 minutes)

1. **Building/Updating PCC/ACP:** How are Shared Care Plans/Advance Care Plans developed or updated? Who facilitates this process? How are urgent situations handled? How clear are directives regarding “no intubation”, NIV, PEG, palliative sedation?

### D. Multidisciplinary Meetings (MDT) (≈6 minutes)

1. **Frequency, participants and typical agenda.**
2. **Impact on coordination and timing:** How do MDTs affect coordination and timing of actions (identifying the owner of each action, deadlines, follow‑up)?

### E. Acute Events/Critical Incidents (≈6 minutes)

1. **Describe a recent episode** in an ALS patient that was well managed: what worked?
2. **Describe a challenging episode:** what was lacking (information, people, tools)?

### F. Barriers/Facilitators (≈4 minutes)

1. **Main barriers:** organisational, cultural, medico‑legal, IT.
2. **Three facilitators** to preserve in the Modena model.

### G. Improvements (≈3 minutes)

1. **Two concrete, feasible changes** for the next 6–12 months.

**Closing (≈30 seconds)** – Anything else to add? May we contact you again for clarifications?

## Specific Modules by Professional Profile

### 1. Neurologists (ALS Centre)

- **Triggers for Palliative Care/MDT:** When do you refer patients to Palliative Care? What clinical or organisational triggers (ALSFRS‑R score, NIV, caregiver distress) do you use?
- **From decision to action:** How do you translate decisions (e.g., “no intubation”, possible palliative sedation) into a 118 warning, PCC updates, or community activations (who does what, by when)?
- **Devices and end of life:** How do you integrate technical choices (NIV/invasive ventilation, PEG) with goals of care? How do you document limits of escalation and communicate these to emergency services/Home Palliative Care?
- **Hand‑over to Home Palliative Care/General Practice:** What are facilitators or obstacles in the handover from clinic to home?

### 2. Home Palliative Care (UCP) / Hospice

- **118 Warning:** Practical criteria for activation; essential contents of the action card; perceived usefulness in the field; follow‑up after a 118/ER intervention.
- **PCC/ACP for emergencies:** Clear formulations (e.g., “no intubation”, palliative sedation); medication kits and caregiver instructions.
- **Hospice and timing:** When do you consider admission? What are the obstacles to “getting the timing right”?
- **MDT as an operational engine:** Provide a concrete example where an MDT meeting led to rapid activations that changed the course of care.

### 3. General Practitioners (MMG) / Interpares

- **Detecting needs at home:** Practical signals (dyspnoea, loss of autonomy, caregiver burden).
- **After‑hours:** How are evening/night/weekend calls handled? What instructions are given for calling 118? How does this integrate with community nursing?
- **Warning and respiratory crisis:** Perceived differences with and without the 118 warning; examples.
- **Participation in MDTs:** Usefulness and barriers (scheduling, remote participation, concise reports).

### 4. Territorial Emergency Services (118 – Doctors/Nurses/Control Centre)

- **Receiving/Using the warning:** Where do you see key information (PCC, “no intubation”)? How complete does it seem?
- **On‑site decisions:** Practical criteria for choosing between intubation and palliative sedation with or without a warning; examples.
- **Handover and coordination:** Contact with Home Palliative Care or Integrated Home Care before leaving the home; IT/logistical obstacles.
- **Training:** What has been useful so far? What training needs remain (on ALS/end of life care)?

## Operational Tips for the Interviewer

- **Elicit at least one critical incident** during each interview.
- **Maintain neutrality:** Focus on processes rather than personal judgements about colleagues or services.
- **Always note** who does what, when and with what information.
