# Supplementary material for: Integrating Neurology, Palliative Care and Emergency Services in ALS: A Community-Integrated Neuropalliative Pathway in Modena, Italy
Source: Brain Sci. 2025 Nov 30;15(12):1294. doi: 10.3390/brainsci15121294 (PMC12730195; doi:10.3390/brainsci15121294)
Supplement: Supplementary file 1 [file brainsci-15-01294-s001.zip › File 2 reflexivity statement.docx]

**Reflexivity Statements**

Sofia Bonilauri:

I am a 28-year-old Caucasian woman, a medical doctor in training in Palliative Medicine, with previous clinical experience in oncology–hematology and geriatrics. Early exposure to vulnerable contexts — including a research period in Egypt and volunteer work in a refugee camp in Thessaloniki — strengthened my sensitivity toward dignity, autonomy, and cultural complexity in serious illness. I am also enrolled in a Master’s program in Bioethics, which has shaped my interest in the ethical and relational dimensions of end-of-life care.My professional values strongly align with the principles underpinning the Modena ALS pathway, particularly early palliative care activation, interprofessional integration, and shared care planning. This alignment likely predisposed me to a positive interpretive bias, highlighting strengths of the model and privileging narratives focused on protection of patient preferences. As a novice qualitative researcher, my limited experience may also have influenced how I approached data interpretation.To minimise these influences, I engaged in reflexive writing, regular discussion within the research team, and intentional attention to divergent perspectives — particularly those expressing discomfort, tension in family dynamics, or uncertainty in emergencies. Triangulation of interviews with field notes from outpatient observations further supported a more balanced interpretation. Making my positionality explicit aims to enhance transparency and contextualize the interpretive lens through which this analysis was developed.

Alberto Canalini:

I am a 41-year-old Caucasian male nurse with a long-standing professional and personal interest in emergency medicine. My clinical career has always been rooted in this field: I worked for four years in the Intensive Care Unit and have been serving for the past thirteen years in the pre-hospital emergency system (118). Over the years, I have participated in several research projects—some as principal investigator and others as collaborator—mainly focused on pre-hospital emergency care, particularly in the areas of cardiac arrest management and major trauma.

Throughout my professional experience, I have encountered patients affected by amyotrophic lateral sclerosis (ALS) in two distinct care settings. The first was during my time in the Intensive Care Unit, where patients required vital function support; the second was in the pre-hospital emergency context, where the disease often reaches its terminal stage and both patients and their families rely on emergency services to manage acute episodes, such as respiratory crises.

These experiences have shaped my perception of the need for an increasingly comprehensive and person-centered approach to patient care—one that supports and guides individuals through the difficult decisions that their condition entails. I believe that the healthcare system’s capacity for integrated care and continuous support represents a valuable resource for professionals, patients, and their families alike. I’m aware of a personal positive bias toward this case study that we are describing, as I hope that it might help patients and families to live and die more according to their preferences.

Gianfranco Martucci:

I am a 40-year-old Caucasian male with a socially respected professional background as a physician, although my specialisation—community medicine, and particularly palliative care—is still relatively uncommon and not always well understood. Throughout my career I have divided my time between clinical work, project development, and research, which has given me an atypical professional profile. My research experience has mainly focused on organisational, educational, and qualitative approaches.

I often find myself balancing two needs: on one hand, the wish to narrate cases that I find meaningful from my professional perspective—stories that may offer insights to others facing similar challenges in their services—and on the other hand, the need to frame these experiences in a way that is of interest to the broader scientific community, which tends to privilege quantitative paradigms.

I have extensive experience in palliative care (seven years of research and five additional years combining
