# Supplementary material for: Integrating Neurology, Palliative Care and Emergency Services in ALS: A Community-Integrated Neuropalliative Pathway in Modena, Italy
Source: Brain Sci. 2025 Nov 30;15(12):1294. doi: 10.3390/brainsci15121294 (PMC12730195; doi:10.3390/brainsci15121294)
Supplement: Supplementary file 1 [file brainsci-15-01294-s001.zip › File 3 Integrated Neuropalliative Care Attachments.docx]

# Integrated Neuropalliative Care Attachments

## Vignettes Illustrating the Integrated Pathway – table 1

| Stage | Patient vignette | Provider/family perspective |
| --- | --- | --- |
| Diagnosis and referral to integrated pathway | Giulia, 58, is diagnosed with ALS at the neuromuscular clinic. After a few months, her ability to walk is severely impaired, and she asks to have a conversation on how things are going to be in the future and what are her option. The neurologist explains the prognosis but also introduces the Community‑Integrated Neuropalliative Pathway. Giulia learns that palliative care is not just for the end of life. She is offered an early consultation and told about the option to activate a shared care plan and the warning system when she feels ready. | The neurologist remains Giulia’s primary point of contact and schedules a multidisciplinary meeting to discuss her needs.Family members are relieved to know there is a coordinated pathway connecting home care, neurology and emergency services, and that decisions about interventions (e.g., PEG, NIV) can be discussed proactively. |
| Home palliative activation and needs assessment | Months later, Giulia experiences increasing fatigue and finds swallowing difficult. Her neurologist contacts the palliative team. A home nurse visits and performs a needs assessment. Giulia is now formally referred to the local palliative care network (LPCN). Regular home visits and social worker support begin. | The palliative team assesses Giulia’s needs and explains that the home team will coordinate with the rest of the team, included the emergency services if needed. Family is regularly involved in goals-of-care discussion. |
| Shared care planning and warning activation | During a series of meetings at home, Giulia, a palliative doctor and the home nurse discuss her values and preferences. They talk about her wishes regarding intubation, resuscitation and hospital admission. Often, her neurologist is present too After several conversations, Giulia signs a Shared Care Plan that specifies she does not want invasive ventilation and prefers to remain at home if possible. The nurse uploads the plan into the emergency warning system. | Professionals emphasise that advance care planning is relational and can be revisited over time; the document is not a one-off decision.The family receives training on how to call emergency services and inform them that a warning flag exists. They feel empowered by the process. |
| Emergency event with warning | Giulia develops respiratory distress at night. Her partner calls 118 and mentions that there is a warning flag.The dispatcher sees the flag, reads Giulia’s shared care plan and informs the ambulance crew. Paramedics arrive with action cards indicating ‘do not resuscitate’ and ‘no hospital admission’. They provide symptom relief at home. | Emergency clinicians appreciate having clear instructions and avoid defaulting to invasive ventilation. Giulia’s family is relieved that the emergency response aligns with her wishes and that she can remain at home. |
| Emergency event without prior palliative flag | Luca, another ALS patient, has never been referred to the palliative team. He visits the ER for severe dyspnea. ED staff uses the VAPU screening tool and identify palliative care needs. He is stabilised, and upon discharge the ED nurse contacts the LPCN within 48 hours so the palliative team can evaluate him at home. | The ED nurse informs Luca’s caregiver about palliative options and arranges follow-up. This referral pathway reduces the risk of repeat emergency visits and ensures that Luca receives appropriate support. |
| End-of-life care and bereavement support | Giulia’s condition declines further. She and her family reaffirm her wish to remain at home. The palliative team provides intensive home visits, symptom control and psychosocial support. She dies peacefully at home, surrounded by her family. After her death, the team offers bereavement support to her partner. | The home care team coordinates with the general practitioner to manage symptoms and medications. Family members express gratitude that Giulia’s preferences were honoured and that they received support during and after her death. |

## Value tensions – table 2

| Table 2 – Key value tensions in integrated neuropalliative care for ALS in the CINP case study | | |
| --- | --- | --- |
| Value 1 |  | Value 2 |
| **Timing of decision-making** | | |
| Work on advance care planning while the patient can still communicate effectively | VS | Introducing choices too early may cause stress-induced paralysis and global refusal |
| **Respect for autonomy and awareness** | | |
| Avoid steering the patient’s decision | VS | Concern that the patient does not grasp the severe implications of living with PEG and ventilation |
| **Prognostic uncertainty** | | |
| Avoid ‘alarming’ the patient given uncertainty about disease evolution | VS | Unpredictability can lead to rapid survival or communication issues |
| **Life-sustaining interventions** | | |
| The burden of initiating life-sustaining support (e.g., PEG, NIV) | VS | The burden of supporting withdrawal of these supports when they become intolerable |
| **Role of GP** | | |
| Value of GP support in daily management of potentially long-surviving patients | VS | Difficulty for GPs to engage in highly specialised and ethically complex dynamics |
| **Symptom relief and respiratory risk** | | |
| Use of morphine and benzodiazepines for symptom relief | VS | Fear of suppressing respiratory function |
| **Non-invasive ventilation (NIV)** | | |
| Use NIV to palliate symptoms before definitive choices | VS | Starting machine dependence that the patient might not desire after deeper reflection |
| **Place of death** | | |
| Honour the patient’s wish to die in the preferred place | VS | Complexity of symptom management for family caregivers at home |
| **Long-term disability** | | |
| Potentially long phase of severe disability even without life-sustaining devices | VS | Lack of support structures for disabilities of this magnitude (hospice stays limited; nursing homes reluctant) |
| **Emergency interventions vs. reflective deliberation** | | |
| Need for clarity on intervention and legal/ethical protection during emergencies | VS | Inherently slower pace of palliative reflections and deliberation |

## Checklist for Integrated Neuropalliative Care Pathways – table 3

| No. | Domain | Checklist question |
| --- | --- | --- |
| 1 | Leadership & Roles | Who initiates palliative care involvement (e.g., neurologist, palliative physician, general practitioner)? |
| 2 |  | Is there a defined case manager across the continuum of care? |
| 3 |  | Are there dedicated coordinators or nurses responsible for connecting home palliative care with emergency services? |
| 4 |  | Are emergency department (ED) professionals identified as palliative care champions or trained to provide primary palliative care? |
| 5 | Integration & Coordination | Are there regular multidisciplinary meetings (e.g., every three months) involving neurology, palliative care, home care and emergency services? |
| 6 |  | Is there a formal mechanism (e.g., ‘warning 118’ or alert) to share advance care plans and patient preferences across hospital, community and emergency services? |
| 7 |  | Does the emergency department use a screening tool (e.g., VAPU) to identify patients with palliative care needs when not previously known to palliative services? |
| 8 |  | Are there clear procedures for ED staff to notify home palliative care teams upon discharge or admission? |
| 9 |  | Are referral pathways between ED and the local palliative care network well-defined and documented? |
| 10 |  | Does the pathway align with regional/national clinical pathway (PDTA) guidelines? |
| 11 | Advance Care Planning | Is advance care planning viewed as a relational, iterative process rather than a one-time form? |
| 12 |  | Do patients and families have multiple meetings to develop and revisit their shared care plan? |
| 13 |  | Is the final care plan available to emergency systems for consultation (e.g., the warning 118) to ensure concordance during crises? |
| 14 |  | Does the care plan explicitly capture patient wishes regarding resuscitation, intubation, hospital admission and palliative sedation? |
| 15 |  | Are patients and families trained on how to contact the emergency service and communicate their status and preferences? |
| 16 | Education & Culture | Do ED professionals receive structured training in palliative care, including symptom management, communication skills and ethical issues? |
| 17 |  | Are there joint educational initiatives to build a shared culture of palliative care among the different professionals involved in assistance, as neurology, palliative, home care and emergency teams? |
| 18 |  | Are mechanisms in place to address bureaucratic delays, logistical challenges and potential resistance from practitioners? |
| 19 |  | Does your service’s training include non-cancer diagnoses like ALS, acknowledging their unpredictable trajectories? |
| 20 |  | Is there a continuous quality improvement process to gather feedback from staff and adapt the pathway and training accordingly? |
| 21 |  | Did you adopt an interprofessional and shared vision when developing the clinical pathway? Approaches such as action research or participatory research may be appropriate. |
| 22 | Quality Monitoring & Outcomes | Are indicators monitored (e.g., number of patients referred from ED to palliative care, number of flagged patients, place of death) to evaluate integration outcomes? |
| 23 |  | Is patient and caregiver satisfaction with the integrated pathway regularly evaluated? |
| 24 |  | Are end-of-life outcomes (e.g., home versus hospital death) recorded and used for quality improvement? |
| 25 |  | Are data and outcomes shared with stakeholders to promote accountability and improvements? |
| 26 |  | Is there flexibility to adapt the pathway to local contexts, recognising that no ‘one-size-fits-all’ model exists? |
